# Workflow for Criticality Assessment Applied in Biopharmaceutical Process Validation Stage 1

**DOI:** 10.3390/bioengineering4040085

**Published:** 2017-10-12

**Authors:** Thomas Zahel, Lukas Marschall, Sandra Abad, Elena Vasilieva, Daniel Maurer, Eric M. Mueller, Patrick Murphy, Thomas Natschläger, Cécile Brocard, Daniela Reinisch, Patrick Sagmeister, Christoph Herwig

**Affiliations:** 1 Exputec GmbH, Mariahilferstraße 147, 1150 Vienna, Austria; thomas.zahel@exputec.com (T.Z.); lukas.marschall@exputec.com (L.M.); patrick.sagmeister@exputec.com (P.S.); 2 Boehringer Ingelheim RCV GmbH & Co KG, Doktor-Boehringer-Gasse 5-11, 1120 Vienna, Austria; sandra.abad@boehringer-ingelheim.com (S.A.); elena.vasilieva@boehringer-ingelheim.com (E.V.); daniel.maurer@boehringer-ingelheim.com (D.M.); cecile.brocard@boehringer-ingelheim.com (C.B.); daniela.reinisch@boehringer-ingelheim.com (D.R.); 3 Versartis Inc., 4200 Bohannon Drive, Suite 250, Menlo Park, CA 94025, USA; emueller@versartis.com (E.M.M.); pmurphy@versartis.com (P.M.); 4 Software Competence Center Hagenberg, Softwarepark 21, 4232 Hagenberg, Austria; Thomas.Natschlaeger@scch.at

**Keywords:** retrospective power analysis, process characterization study, process validation stage 1, criticality assessment, control strategy, design of experiments

## Abstract

Identification of critical process parameters that impact product quality is a central task during regulatory requested process validation. Commonly, this is done via design of experiments and identification of parameters significantly impacting product quality (rejection of the null hypothesis that the effect equals 0). However, parameters which show a large uncertainty and might result in an undesirable product quality limit critical to the product, may be missed. This might occur during the evaluation of experiments since residual/un-modelled variance in the experiments is larger than expected a priori. Estimation of such a risk is the task of the presented novel retrospective power analysis permutation test. This is evaluated using a data set for two unit operations established during characterization of a biopharmaceutical process in industry. The results show that, for one unit operation, the observed variance in the experiments is much larger than expected a priori, resulting in low power levels for all non-significant parameters. Moreover, we present a workflow of how to mitigate the risk associated with overlooked parameter effects. This enables a statistically sound identification of critical process parameters. The developed workflow will substantially support industry in delivering constant product quality, reduce process variance and increase patient safety.

## 1. Introduction

Process validation of pharmaceutical processes aims to demonstrate the capability of the process to constantly deliver high product quality [1,2]. Most of the warning letters connected to process validation are raised due to flaws in stage 1 [3]. The aim of process validation stage 1 is to identify a robust process design that enables the ability to constantly deliver product quality. Therefore, it is key to identify critical process parameters (CPPs) that are likely to create risk to critical quality attributes (CQAs) and set up control strategies for these CQAs. Thereby it is possible to reduce out-of-specification (OOS) events, recalls, and ultimately risk to the patient. At process validation stage 1, it is of the highest priority not to overlook a CPP in the design of the process, which as a consequence might not be controlled properly.

In order to accomplish this goal, the following steps are commonly undertaken in industry to characterize process design following a risk-based approach:

1. Risk assessment: to identify potential influential/critical parameters for each unit operation. This is usually performed using tools such as failure mode and effect analysis (FMEA) [4,5]. Ranking of potential criticality is performed using expert knowledge, historical process data, and interdependencies identified in development data.
2. Scale down model establishment: Due to the costs related to large-scale experiments, in biopharmaceutical manufacturing it is necessary to develop appropriate scale down models (SDMs) that are appropriate to investigate the interdependency between process parameters and quality attributes.
3. Experimental designs: Design of Experiments are applied to quantify the impact of process parameters (PPs) on CQAs. Prior to conducting experiments, a priori power analysis is a good practice to evaluate if an effect that leads to a change in product quality—in the following defined as a critical effect—can be detected by the proposed design setting. Statistical power is defined as the probability that we are able to detect an effect if it is truly there [6]. This is done for a priori analysis by estimating the expected signal to noise ratio, which is thought to occur during the experiments [7]. As a result of this a priori power analysis, the number of required experiments, the intended screening range, or the design itself might be adjusted. After a sufficient power can be expected, potential influential/critical parameters are purposefully varied within experiments, which is done for each unit operation separately using the previously established SDMs.
4. Criticality assessment of process parameters by evaluating experimental designs: Identification of significant factors (rejection of the null hypothesis that the effect equals 0) at a desired significance level (typically *α* < 0.05) is performed using Pareto charts and analysis of significance of regression coefficients by means of ANOVA. Misleadingly, this does not imply that for non-significant factors the null hypothesis is true and their effect is zero [8]. Rather, it indicates that the uncertainty around these factors in the range examined—often indicated by large confidence intervals around the effect—is large and critical levels cannot be excluded. Commonly, only significant factors that have been observed to impact product quality or process performance are defined as critical or key, respectively. Those which cannot be stated as significantly impacting are stated as non-critical or non-key, respectively.
5. Definition of control strategy: As a means to ensure all CQAs and quality specifications are met, a process control strategy for all critical and key process parameters must be put in place. Moreover, it has to be evaluated whether their mutual worst case setting would lead to acceptable product quality levels. Commonly for biopharmaceutical production, this is accomplished by setting normal operating ranges (NOR) and proven acceptable ranges (PAR).

Although all steps are equally important to design a robust process, we frequently observed that, in industry, steps 3, 4 and 5 are more difficult to accomplish in practice. The US Food and Drug Administration (FDA) and other agencies are not prescriptive but clearly state that statistics should be used within all stages of process validation [3]. Multiple statistical tools and software for step 3 (a priori power analysis and design of experiments) and step 4 (statistical analysis of significant parameters) exist, however, the approach of those steps as described above has two major drawbacks: (i) after making several assumptions about the expected noise in the a priori power analysis of step 4, those assumptions are not checked for validity after the experiments have been performed. Especially in biopharmaceutical engineering, reproduction and analytical variability from non-validated methods, which might be used during stage 1 of process validation, as well as unexpected non-linear effects (e.g., edge of failure experiments), may lead to increased noise in the conducted design of experiments (DoEs). (ii) Criticality and potential tightening of the NOR is only taken into account for significant parameters. This might not be sufficient since parameters with large uncertainty around the estimated effect—those effects, which might be zero, but might be very large, too—can have severe effects on product quality as well.

The first of the mentioned drawbacks can be tackled by retrospective assessment of the actually received power. Although retrospective power analysis is controversially discussed when using the observed variance and observed effect size, it is an appropriate tool when comparing the observed variance in the experiments to a pre-specified critical effect [6,9]. Frequently, retrospective power is calculated using the observed effect size, which leads to uninformative results [10].

Both issues together might lead to situations where the process shows unexpected variability during routine manufacturing. Therefore, we want to present a workflow for criticality assessment that reduces the risk to overlook critical PPs. This is demonstrated based on a process characterization study of a novel long acting human growth hormone product. Exemplarily for two unit operations, we will address the following topics:

- Establishment of a methodology that prevents engineers, during process validation, from overlooking critical parameters;
- Setting a control strategy for critical and likely overlooked parameters that ensures a robust process design;
- A workflow that can be used during stage 1 process validation to assess PP criticality. Applying those guidelines, it will be possible to better understand potential process variability and provide an opportunity to reduce process variability, OOS events, and patient risk.

## 2. Methods

In the following sections, we describe the biopharmaceutical production process, selection of experimental designs to study the impact of PPs on CQAs (Section 2.1), calculation procedures for critical effects (Section 2.2), an a priori power analysis approach (Section 2.3) applied to assess the ability of the DoE to detect practically relevant (here critical) effects and their statistical evaluation (Section 2.4). 

### 2.1. Description of Process and Design of Conducted Experiments

The workflow for criticality assessment will be presented for two unit operations from a biopharmaceutical manufacturing process producing a recombinant protein. The process consists of an Escherichia coli fermentation, cell lysis, precipitation (PR), clarification (depth filtration), and three subsequent preparative chromatographic columns (CC 1/CC 2/CC 3) for purification. Finally, ultrafiltration/diafiltration is performed to adjust product concentration. For the presented case study for criticality assessment, unit operations CC 1 and the precipitation step were exemplarily chosen. 

Risk assessment (FMEA) conducted by process experts showed that five and four PPs respectively, had a high risk priority number and need to be studied experimentally in respect to their influence on CQAs for CC 1 and PR, respectively (see Table 1 and Table 2). Due to the number of studied PPs for both unit operations, a definitive screening design was chosen [11,12]. Except one parameter (Mixing [Yes/No] for precipitation), all DoE factors are numerically scaled. Small-scale experiments were used to conduct DoEs.

### 2.2. Calculation of Thresholds for Critical Effects 

We formulate a critical gap (CG) as the difference between the performance at set-point conditions and the threshold for each response:

(1) $$\mathrm{CG}={\mathrm{threshold}}_{\mathrm{USL}}-\bar{y}\left(x_{SP}\right)$$

where $\bar{y}\left(x_{SP}\right)$ is the response value (here a specific concentration of an impurity) at set-point condition of manufacturing. Since we do not have lower specification limits for the studied impurities, the threshold, which must not be surpassed, is derived from the upper specification limit (USL) of drug substance (DS) specifications. The studied unit operations are at an intermediate stage of the process. We therefore, calculate the specification limit for the investigated unit operation by multiplying the final DS specifications times the mean specific clearance factors from the manufacturing scale of all unit operations in between. This approach might be refined by including knowledge on increased impurity clearance, e.g., due to spiking studies. Choosing the approach with mean specific clearances might seem conservative, however, it is desirable to reduce the risk of overestimating the specific impurity clearance. The specific clearance factors for each unit operation are defined by:

(2) $$\mathrm{Specific}\;\mathrm{Clearance}=\mathrm{SC}=\frac{c_{\mathrm{CQA},\mathrm{load}}}{c_{\mathrm{CQA},\mathrm{pool}}}$$

where cCQA, load and cCQA, pool are the specific concentrations (mg CQA per mg product) of the respective CQA prior to and after the unit operation.

(3) $${\mathrm{threshold}}_{\mathrm{USL}}=\;\mathrm{USL}*\overset{U}{\underset{u=k}{\prod }}{\mathrm{SC}}_{u}$$

where *u* = *k*, …, *U* is counting the unit operations from the studied *k*th unit operation until the last unit operation (*U*) which equals DS.

### 2.3. A Priori Power Analysis

We want to investigate if the residual error during evaluation of experimental designs (DoEs) masks effects to an extent such that they could collectively surpass a critical threshold (e.g., specification limit of a specific CQA concentration) within normal operating ranges (see Section 2.2 for calculation of thresholds). Since we are dealing with a multivariate problem, we need to identify how many parameters and to what extent each of those parameters contributes to surpassing such a critical threshold. From a sparsity assumption, it is unlikely that all effects that can be studied using a certain design (e.g., all main effects and interactions effects) are truly present. Therefore, it is a common assumption applied to many statistical packages to study only power of the total number of main effects [13]. 

Moreover, in multivariate analysis (*p* > 1), infinite combinations of effects of multiple parameters exist that lead to such a critical threshold being surpassed, e.g., the full effect to surpass the critical threshold might be explained solely by the first parameter ($P_{1}$) and no effect is present from the residual parameters ($P_{r}$), or a fraction of the entire effect is explained by P1 (e.g., 10%) and the residual 90% is equally explained by $P_{r}$. Overall, we are interested in the mean chance to detect any of those combinations. Per default, classical statistical software such as JMP (SAS Institute Inc., Cary, NC, USA) or DesignExpert (Stat-Ease, Inc., Minneapolis, MN, USA) only allow for fixed effect power calculation [10,13]. Here, we propose a more general method based on the assumption that the effects are randomly distributed over all parameters. Therefore, we assigned weights to the parameters and varied the fraction/weight of the entire effect that is explained by each parameter gradually between 0.0 and 1.0 (we used a step size of 0.01 in our experiments, i.e., 100 steps) and split the residual effect equally under the residual parameters: $w_{i}=a$, $w_{j\neq i}=\left(1-a\right)/\left(p-1\right)$, for a=0, …,1 and i=1,…, p. Hence all the weights $w_{i}$ sum up to 1. In total, we obtain C=p∗100 combinations of possible effect distributions and the resulting power values. The mean for each parameter of these recorded power values was taken as the power for this experimental design (see step 6 of the a priori workflow present below). 

Herein, the following workflow for a priori power analysis can be formulated:

Estimate the mean (${\bar{y}}_{SP}$) and variance ($\sigma_{SP}$) of the response variable from small-scale or pilot-scale experiments at set point conditions of manufacturing. We assume that residual error in the model is only due to process- and analytical variance. The latter estimate will be used to calculate the expected sum of squares of the residuals ($\tilde{SS_{res}}$):
  (4) $$\tilde{SS_{res}}=\left(n-1\right)*\sigma {²}_{SP}$$
For each of the combinations (*c*) described above, we calculate critical effects for each parameter using its weight $w_{i}^{\left(c\right)}$:
  (5) $$\beta^{\left(c\right)}{}_{crit,i}=\frac{w_{i}^{\left(c\right)}*\mathrm{CG}}{\max\left({\mathrm{NORU}}_{i}-sp_{i},sp_{i}-{\mathrm{NORL}}_{i}\right)}$$
  In order to estimate the individual coefficient for the *i*-th parameters, from a risk-based approach, we divide by the longest distance from the set-point ($sp_{i}$) to the nearest NOR border: where ${\mathrm{NORU}}_{i}$ is the upper boundary of the NOR and ${\mathrm{NORL}}_{i}$ is the lower boundary of the NOR of the parameter *i*. Note that this works for a symmetric as well as asymmetric NOR.
Using the design matrix X, obtained for a specific experimental design, we can simulate possible $\tilde{y}$ values at the screening range using:
  (6) $${\tilde{y}}^{\left(c\right)}=X\beta_{crit}^{\left(c\right)}$$
From that, the total sum of squares can be estimated:
  (7) $$\tilde{SS_{tot}{}^{\left(c\right)}}=\overset{n}{\underset{i}{\sum }}\left({\tilde{y_{i}y}}^{\left(c\right)}-mean\left({\tilde{y}}^{\left(c\right)}\right)\right)²$$
  Together with the sum of squares of the residuals, the expected coefficient of variance can be calculated:
  (8) $${\tilde{R}}^{2}{}^{\left(c\right)}=1-\frac{\tilde{SS_{res}}}{\tilde{SS_{tot}{}^{\left(c\right)}}}$$
Using Cohen’s effect size (f), the non-centrality parameter *λ* and the critical *F* value ($F_{crit}$), the a priori power for the combination *c* of effects that no parameter has been overlooked can be calculated [7]:
  (9) $$f^{2^{\left(c\right)}}=\frac{{\tilde{R}}^{2^{\left(c\right)}}}{1-{\tilde{R}}^{2^{\left(c\right)}}}$$
  (10) $$\lambda^{\left(c\right)}=f^{2^{\left(c\right)}}*\nu$$
Confidence intervals for the a priori power for the combination *c* were calculated according to
  (11) $$\lambda_{\mathrm{upp}}^{\left(c\right)}=\lambda^{\left(c\right)}*c_{crit}(1-\alpha |\nu )/\nu$$
  (12) $$\lambda_{low}^{\left(c\right)}=\lambda^{\left(c\right)}*c_{crit}(\alpha |\nu )/\nu$$
  where $c_{crit}\left(\alpha |\nu \right)$ is the 100∗α percentile from a χ^2^ distribution with ν degrees of freedom.
  (13) $$F_{crit}=F_{inv}\left(1-\alpha |\;u,\nu \right)$$
  (14) $$power_{apriori}{}^{\left(c\right)}=1-F_{nc}\left(F_{crit}|,\;u,\nu ,\lambda^{\left(c\right)}\right)$$
  where $F_{nc}$ is the non-central *F* distribution with u=p (number of DoE parameters) and ν=n−u−1, where *n* is the number of observations in the DoE.
The mean power over all combinations of effects was estimated as the arithmetic mean of all $power_{apriori}{}^{\left(c\right)}$:
  (15) $$power_{apriori}=\frac{\sum_{c=1}^{C}power_{apriori}{}^{\left(c\right)}}{C}$$

### 2.4. Evaluation of DoEs 

Multiple linear models were used to identify the relationship of the studied PPs (DoE factors, *X*) on the response variable (*y*), representing a CQA or KPI of the process, up to a residual error (*ε*):

(16) $$y=\;\beta_{0}+X\beta +\varepsilon$$

where *X* is a (*n* × *p*) dimensional design matrix for n DoE runs and p DoE factors which are studied, $\beta_{0}$ is the intercept, β are the true effects of the DoE factors, and ε is the residual, un-modelled error vector. The un-modelled error vector describes the analytical and process variance as well as non-linear effects which cannot be accounted for in the model structure. Identification of significant parameters was done using stepwise regression within the multiple linear regression (MLR) tool of inCyght software (inCyght version 2017.03, Exputec GmbH). Parameters showing a partial *p*-value below 0.05 were allowed to enter the model. Those which showed a *p*-value larger than 0.1 were excluded from the model. Starting with the most significant parameter, this including/excluding procedure was applied iteratively and was repeated till the model structure did not change any more and the optimal model was achieved by this approach; identified significant parameters and their respective *p*-value are shown in Table 1 and Table 2 for CC 1 and PR, respectively. The normalized raw data are given in the Supporting Information Tables S1 and S2.

## 3. Results and Discussion

Experiments performed in biotechnological studies might contain data that violate the statistical assumptions of parametric tests (i.e., normality, homogeneity of variances and independence of errors). Moreover, with a limited number of experiments and a large number of unknown parameters, such assumptions are hard to assess. Consequently, nonparametric approaches bear potential and we want to present a novel permutation test to assess the power of individual DoE factors in a multivariate regression model. 

### 3.1. Permutation Test for Retrospective Power Analysis

The following permutation approach is adapted from a permutation test aiming to investigate power retrospectively [14]. Here, we adapted this approach to study the significance of the alternative hypothesis that critical effects are present. The following steps are performed:

1. Using variable selection procedures, we select a significant regression model (all included effects are not 0 to a certain significance level):
  (17) $$y=\;\beta_{0}+\beta_{s}*X_{s}+R_{y|X_{s}}$$
  where $X_{s}$ denotes the s significant parameters selected from a variable selection procedure (e.g., stepwise variable selection) and $R_{y|X_{s}}$ are the residuals of the obtained model. A list of those significantly selected parameters for the case studies of this work can be found in Table 1 and Table 2.
2. We define a critical gap (CG) that we must not surpass as the difference of the threshold and the worst case model prediction within the NOR ($x_{worst\;case_{\mathrm{NOR}}})$, which is the parameter setting where the model prediction ($\hat{y}\left(x\right)$) is closest to the ${\mathrm{threhsold}}_{\mathrm{USL}}$:
  (18) $$\mathrm{CG}={\mathrm{theshold}}_{\mathrm{USL}}-\hat{y}\left(x_{worst\;case_{\mathrm{NOR}}}\right)$$
3. Similar to the approach discussed in Section 2.3 for the a priori power analysis, for non-significant parameters, a variety of combinations (in total C) of effects for those parameters exist that lead to surpassing a critical threshold. In order to estimate the mean likelihood of not overlooking a specific parameter, we vary the relative impact on the threshold of each parameter gradually between 0 and 1 in 100 steps. The fraction of the CG which is attributed to the non-significant parameter i is expressed as the weight $w_{i}^{\left(c\right)}$ for the combination *c*. Equation (5) can be used to calculate the critical effect of the parameter i.
4. The residuals $R_{y|X_{s}}$ are permuted randomly, producing $R^{*}{}_{y|X_{s}}$.
5. New response values are calculated from the permuted residuals assuming that the critical effect is present under the alternative hypothesis ($H_{A}$):
  (19) $$y^{*}=\;\beta_{0}+\beta_{s}*X_{s}+\beta^{\left(c\right)}{}_{crit}*Z+\;R^{*}{}_{y|X_{s}}$$
  where $\;\beta^{\left(c\right)}{}_{crit}$ is a vector of regression coefficients for the non-significant parameters and Z is the design matrix for all non-significant parameters.
6. Make a model for $y^{*}\;$ based on *X* and *Z* and record significance of ${\hat{\beta }}_{crit}$ at a certain significance level (here *α* = 0.05)
7. Repeat steps 4, 5 and 6 a large number of times (here 1000) and count the number of significant outcomes for each ${\hat{\beta }}_{crit,i}$ at a certain significance level (here *α* = 0.05). The fraction of significant outcomes of all iteration cycles equals the retrospective power of parameter i.

### 3.2. Comparison of a Priori and Retrospective Power

If we apply the proposed retrospective power analysis permutation test of Section 3.1 to experimental data recorded from two unit operations (CC 1 and PR), we obtain power values for each PP/CQA combination from Table 1 and Table 2, respectively.

**Figure 1:**
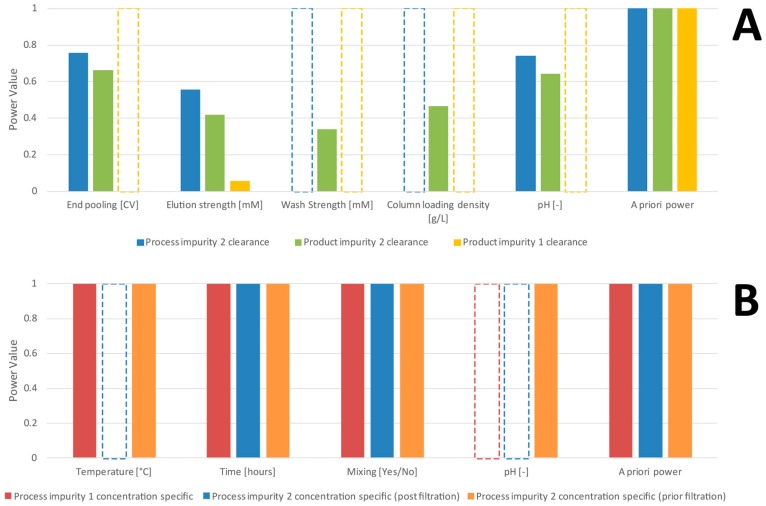
Power values for chromatographic column (CC) 1 (**A**) and PR (**B**) for each process parameter (PP) and CQA. Where significant process parameters were detected for a quality attribute, bars are marked grey. (**A**) Though a priori power analysis suggested a power of 100% for each investigated CQA for chromatography step 1, retrospective power analysis revealed that the power to detect a critical effect did not surpass 80% for any of the investigated process parameters. Strategies to tackle these low-power-situations are given in Figure 4. (**B**) For the precipitation step, a priori power analysis suggested a power of 100% for each investigated CQA as well. Retrospective power confirmed the findings that there is a 100% chance that we did not overlook a critical effect of the investigated process parameters on quality attributes.

Figure 1A shows a comparison of the retrospective and a priori power analysis for the CC 1 unit operation. For all three studied CQAs at this stage (‘process impurity 2 clearance’, ‘product impurity 2 clearance’ and ‘product impurity 1 clearance’), we obtain a priori estimates of 1 (rightmost bar group in Figure 1A). This indicates an ideal case to start with experiments since there is no chance of overlooking a critical effect. Retrospective power analysis revealed that all investigated PPs power values are well below the common statistical practice cut-off value of 0.8. This can be explained by the fact that the residual variance in the model is much higher than the initial estimate at the set point, expressed by ratios of $\frac{{\hat{\sigma }}_{residues}}{{\hat{\sigma }}_{SP}}$ well above 1, as shown in Table 1. In general, multiple reasons for this discrepancy between the initial guess of expected variance and the actual residual variance in the model might exist. It could be a non-representative selection of set-point runs (e.g., runs conducted with different operators), unexpected increase of variance during experiments (e.g., it is more difficult to control experiments at unusual parameter settings) or even non-linear dependency which cannot be captured by the linear model structure. Although statistically good practice, our experience shows that such non-linear dependencies might not be obvious from analysis of residuals (e.g., investigation of plots of residual vs. DoE factors). In a DoE approach, each experiment is unique in its settings if we do not use replicates and thereby no redundancy is available to hinder the model from being leveraged by non-linear responses.

For the precipitation step (PR), a priori power analysis again suggested a power of 1 (Figure 1B). Retrospectively assessed power values match the results obtained from a priori analysis, indicating that the performed DoE had sufficient power to assess critical effects of process parameters on quality attributes. This is reasonable since ratios of $\frac{{\hat{\sigma }}_{residues}}{{\hat{\sigma }}_{SP}}$ are closer to 1 for this unit operation compared to CC 1, as shown in Table 1.

### 3.3. How to Deal with Low-Powered Parameters?

**Figure 2:**
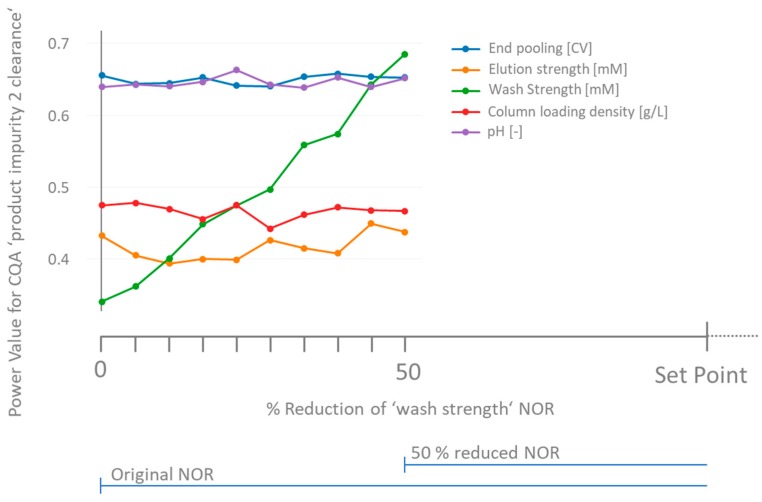
Retrospective power values for ‘product impurity 2 clearance’ for unit operation CC 1 as a function of tightened NOR of process parameter ‘wash strength’. At the initially defined NOR, the power value is 0.34. Upon reducing the NOR symmetrically by 50%, the power value for this process parameter increases to 0.68. The power values of the residual process parameters remain unaffected. The visible variation can be attributed to the variance in the permutation test.

The most common approaches to tackle insufficient power values in screening designs are by increasing the sample size, reduction of measurement variance (either analytical or process), increasing the screening range if technically possible, or accepting the lack of power, however stating the parameter as key or critical. The latter strategy will have an impact on the extended monitoring of such parameters during a subsequent process performance qualification (PPQ) campaign and routine manufacturing. As seen in Section 3.2, a priori power analysis suggested high power values for all investigated unit operations, however, drastically overestimated the power for CC 1. In specific cases, retrospectively increasing the sample size or the screening range might not be possible due to shortage of starting material or technical limitations. A measurement method with less variance might not be at hand to re-measure backup samples. Another approach made possible by the presented method for power analysis is to narrow the NOR of some process parameters. If the threshold stays the same and the NOR is symmetrically located around the set point, for smaller NORs larger effect sizes are necessary to surpass the critical threshold as shown in Equation (5) (i.e., steeper slopes). As a demonstrating scenario, we have chosen the relatively low power for Product impurity 2 clearance on CC 1 (see Figure 1A). For this response, no significant parameter could be found. Figure 2 shows how a reduction of the NOR of the process parameter, ‘wash strength’, impacts the power of all studied PPs of this unit operation. Upon reducing the initially defined NOR by 50% of its width, the power value for ‘wash strength’ increases from 0.34 to 0.68. As seen in Figure 2, the power values of the residual process parameters’ effects on the same quality attribute remained unaffected, neglecting the residual variation caused by the Monte Carlo approach in permutation. 

**Figure 3:**
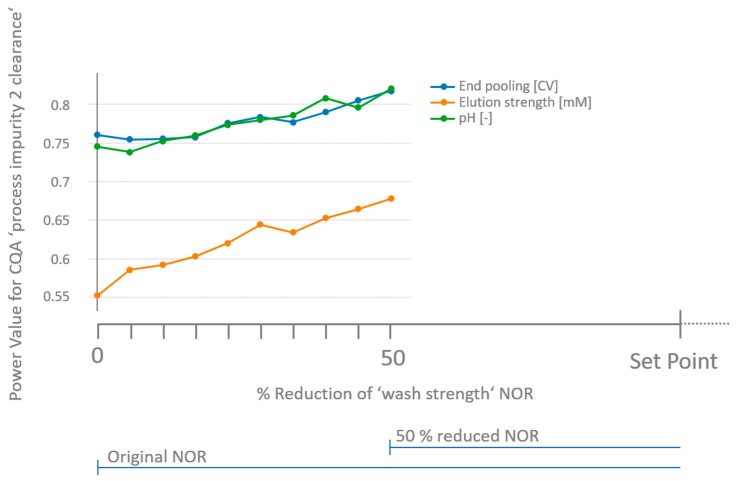
Retrospective power values for ‘process impurity 2 clearance’ for unit operation CC 1 as a function of tightened NOR of process parameter ‘wash strength’. Since wash strength and column loading density are significant parameters in this model, the power was not assessed for those two parameters. Upon reducing the NOR symmetrically by 50% of the significant parameter ‘wash strength’, power values of all other parameters increase since the critical gap is increased, too, due to a reduction of the worst case model prediction in the NOR (Equation (18)).

This provides an opportunity to implement a tighter control strategy though adjusting the NOR as an approach to ensure no critical effects have been overlooked. However, it may not be technically feasible or desirable for all process parameters to implement a tighter control strategy with narrower ranges, especially for a parameter that has not been confirmed to significantly impact a CQA. Since a process parameter is studied in respect to multiple CQAs, we want to note that the tightening of a NOR of a process parameter that significantly impacts one specific CQA will also increase the capacity to not overlook this parameter regarding all other CQAs which have been studied in the same experiment. In contrast to changing the NOR of a non-significant parameter onto a CQA as shown for the combination ‘wash strength’ onto ‘product impurity 2 clearance’ in Figure 2, we investigated how the change of a significant parameter impacts power levels (Figure 3). This was exemplarily done for a decrease in NOR of ‘wash strength’ and we recorded power values for ‘process impurity 2 clearance’ of all non-significant parameters as (here End pooling, elution strength and pH), as shown in Figure 3. We can see that due to the reduction of the NOR of a significant parameter, the power values of all non-significant parameters increase too. In detail, a 50% reduction of the NOR of the significantly impacting parameter ‘wash strength’ increases the power of all non-significant parameters by approximately 10%. This can be explained by the fact that the worst case model prediction within the reduced NOR leads to a larger CG as defined in Equation (18). Thereby, the critical effects will also be larger (Equation (6)) and consequently the chances of overlooking larger critical effects will be reduced. In this way, an improved control strategy for a known significant parameter would improve the confidence that all residual non-significant parameters were not overlooked. This is potently a more desirable approach as improved control of known significant parameters is typically required and advantageous, if feasible.

### 3.4. Workflow for Criticality Assessment

**Figure 4:**
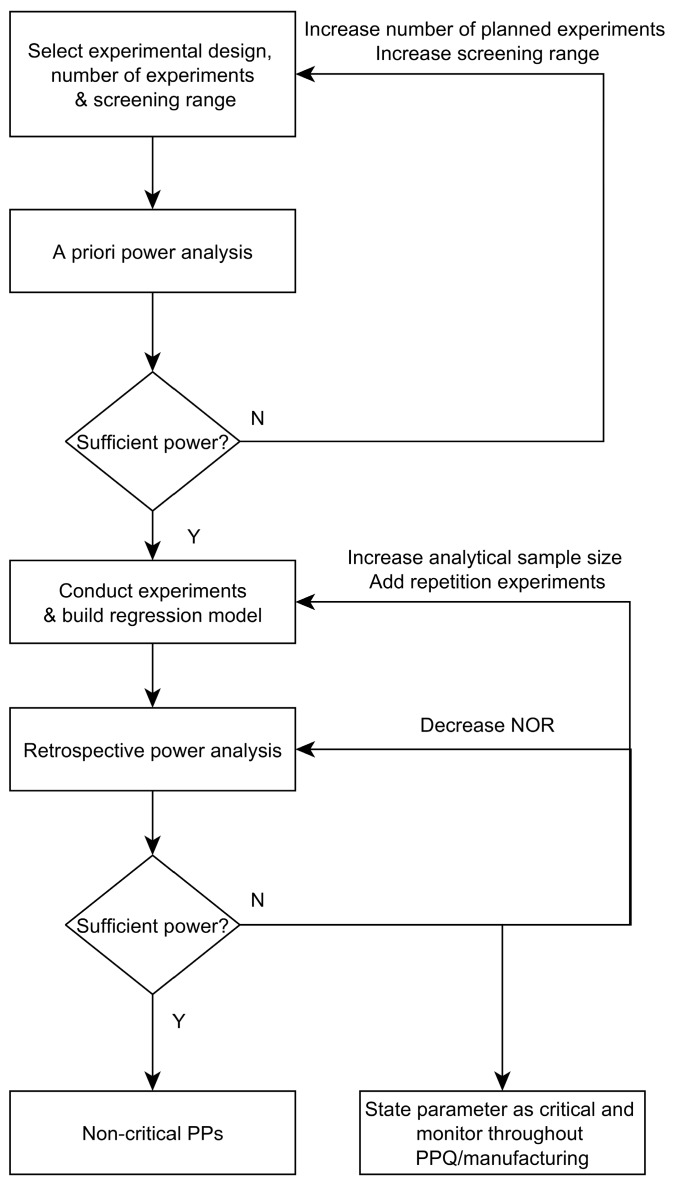
Workflow for criticality assessment of process parameters during process validation stage 1.

In order to summarize the knowledge obtained from the application of the proposed posterior power analysis on two unit operations, we present a workflow that should aid process engineers in assessment of critical parameters (Figure 4). After selection of design and appropriate experiment number, a priori power analysis identifies if it is likely that a critical effect will not be overlooked. Sufficient power levels are normally assumed at 0.8 to 0.9. In cases where sufficient power cannot be assumed, the number of experiments, type of design or screening range must be increased. Both add to the expected signal to noise ratio. When increasing the screening range, care must be taken not to incur failure in experiments due to technical limitations or likely interaction effects (edge of failure). In order to reduce the risk of edge of failure experiments, it is beneficial to conduct an expected worst case scenario of the process parameters first and potentially revise the screening range afterwards.

In case sufficient power can be assumed, experiments can be conducted and regression modelling can be performed together with selection of significant DoE factors/parameters. After the “optimal” model was selected with its significant factors, retrospective power analysis, as shown in Section 3.1, will estimate the chances that the residual non-significant factors might contribute to effects that surpass a pre-specified critical threshold. In case all non-significant parameters show power values well above 0.8 to 0.9, all of them can be stated as non-critical since the residual chance that they have been overlooked is only 20 to 10%, respectively. Otherwise, for those parameters that show insufficient power, analytical and/or reproducibility variance might be lowered by re-measurement of the samples or re-conducting of experiments, respectively. Another option is to narrow the NOR of potentially overlooked parameters which show large variability. This decreases their respective critical effect according to Equation (5). After one of those three countermeasures has been taken, retrospective power analysis can be repeated to ensure sufficient power values are reached and all parameters can be stated as non-critical. If none of the above three options is technically feasible or desirable, potentially overlooked parameters should be stated as critical and monitored during process performance qualification (PPQ) runs or routine manufacturing. 

## 4. Conclusions

The goal of the contribution was to demonstrate the capability of a multivariate retrospective power analysis methodology to identify critical process parameters during pharmaceutical process validation stage 1. 

We have shown in a case study that parameters that are non-significant in models, which were initially thought to be sufficiently powerful to identify critical effects, might still show effects that surpass a critical threshold due to increased analytical, process, or reproducibility variance. This leads to situations where the impact of those parameters on final drug product quality cannot be excluded. This was shown using a biopharmaceutical case study conducted at a world leading CMO. However, common practice is to state such parameters as non-critical and thereby overlook their potential harmful impact. Therefore, two missing parts have been introduced in this contribution: (i) a novel permutation methodology for multiple linear regression that estimates retrospective power (i.e., the chance of non-significant parameters to mutually combine to a critical effect) and (ii) a workflow for criticality assessment that shows strategies of how to mitigate the risk of low-powered parameters. Besides the well-known fact that an increase in experiments increases power, it could be shown that a reduction of the NOR of significant parameters increases the power of all non-significant parameters via a reduction of the worst case model predictions; a reduction of the NOR of a specific non-significant parameter increases power solely for this parameter. Additionally, if implementation of tighter NOR controls is practically infeasible, this methodology can, at a minimum, appropriately assess the process risk and increase awareness of the limitations of the initial classification, potentially suggesting that an improved control strategy is required.

Using both tools, it will be possible for process engineers during the design stage of a process validation (stage 1) to:

- reduce the chance of overlooking potential CPPs
- develop a control strategy for potentially overlooked CPPs in order to increase process robustness
- lower OOS events and finally contribute to increased patient safety.

## Figures and Tables

**Table 1:** *p*-values of significant process parameters that were used in the statistical models for each critical quality attributes (CQA) of CC 1. Normal operating ranges and thresholds are given for each process parameter or critical quality attribute, respectively. Non-significant parameters are indicated with “-”. Also, the ratio of standard deviation of raw residuals of the model by the standard deviation at set-point ($\frac{{\hat{\sigma }}_{residues}}{{\hat{\sigma }}_{SP}}$ is given for each CQA.

		End Pooling [CV]	Elution Strength [mM]	Wash Strength [mM]	Column Loading Density [g/L]	pH [–]	$\frac{{\hat{\sigma }}_{residues}}{{\hat{\sigma }}_{SP}}$
CQA	NOR ^1^	−1.1–0	−1.1–0.65	−1.1–1.1	−0.51–1.1	−0.55–0.55	
	Threshold						
Process impurity 2 clearance	0.85	-	-	0.059	0.099	-	7.79
Product impurity 1 clearance	1.08	0.028	-	0.098	0.089	0.027	18.12
Product impurity 2 clearance	0.1	-	-	-	-	-	256.06

^1^ NOR was normalized by the screening range.

**Table 2:** *p*-values of significant process parameters that were used in the statistical models for each CQA of precipitation (PR). Normal operating ranges or thresholds are given for each process parameter or critical quality attribute. Non-significant parameters are indicated with “-”. Also, the ratio of standard deviation of raw residuals of the model by the standard deviation at set-point ($\frac{{\hat{\sigma }}_{residues}}{{\hat{\sigma }}_{SP}}$ is given for each CQA.

		Temperature [°C]	Time [Hours]	Mixing [Yes/No]	pH [–]	$\frac{{\hat{\sigma }}_{residues}}{{\hat{\sigma }}_{SP}}$
CQA	NOR ^1^	−1.71–0.41	0.33–0.41	−0.95–0.95	−0.61–0.61	
	Threshold					
Process impurity 1 concentration specific	9 × 10^5^	9 × 10^−5^ *	-	-	0.07	64.89
Process impurity 2 concentration specific (prior filtration)	9 × 10^4^	-	-	-	-	2.68
Process impurity 2 concentration specific (post filtration)	784.7	-	-	-	0.021	0.55

^1^ NOR was normalized by the screening range. * A quadratic effect was modelled for temperature and the shown *p*-value corresponds to the quadratic effect.

## Supplementary Materials

The following are available online at www.mdpi.com/2306-5354/4/4/85/s1, Table S1: Standardized experimental data from DoE study of primary recovery (PR), as well as upper and lower normal operating ranges (NOR_U, NOR_L, respectively) and scale down model (SDM) variance and mean. Normalization was performed by subtracting all values by the mean and diving by the standard deviation of DoE runs, Table S2: Standardized experimental data from DoE study of chromatography column 1 (CC1), as well as upper and lower normal operating ranges (NOR_U, NOR_L, respectively) and scale down model (SDM) variance and mean. Normalization was performed by subtracting all values by the mean and diving by the standard deviation of DoE runs.
