# Supplementary material for: Workflow for Criticality Assessment Applied in Biopharmaceutical Process Validation Stage 1
Source: Bioengineering (Basel). 2017 Oct 12;4(4):85. doi: 10.3390/bioengineering4040085 (PMC5746752; doi:10.3390/bioengineering4040085)
Supplement: Supplementary file 1 [file bioengineering-04-00085-s001.pdf]

Supporting Information: Criticality Assessment Workflow for Biopharmaceutical Process Validation Stage 1

Table S1: Standardized experimental data from DoE study of primary recovery (PR), as well as upper and lower normal operating ranges (NOR_U, NOR_L, respectively) and scale down model (SDM) variance and mean. Normalization was performed by subtracting all values by the mean and diving by the standard deviation of DoE runs.

Batches	Parameter: temperature	Parameter: time	Parameter: Mixing [Yes/No]	Parameter: pH	Process impurity 2 concentration specific (post filtration)	Process impurity 1 concentration specific	Process impurity 2 concentration specific (prior filtration)
DoE1	0,00	1,22	0,95	-1,22	-0,88	-1,20	-0,55
DoE2	1,22	-1,22	0,95	-1,22	-0,81	0,80	-0,63
DoE3	-1,22	0,00	-0,95	-1,22	-0,74	0,06	-0,23
DoE4	0,00	-1,22	-0,95	1,22	0,87	-0,88	-0,39
DoE5	-1,22	-1,22	0,95	0,00	-0,71	0,93	-0,41
DoE6	1,22	1,22	-0,95	0,00	-0,76	0,51	-0,66
DoE7	-1,22	1,22	-0,95	1,22	2,07	0,68	-0,24
DoE8A	0,00	0,00	0,95	0,00	0,17	-1,25	1,98
DoE9A	0,00	0,00	-0,95	0,00	0,92	-1,03	1,76
DoE10	1,22	0,00	0,95	1,22	-0,14	1,40	-0,63

Threshold					78,76	24,41	77,31
NOR_L	-1,71	0,33	-0,95	-0,61			
NOR_U	0,41	0,41	0,95	0,61			
Sign Params					Parameter: pH	Parameter: pH, Parameter: temperature	
SDM_variance					0,99	0,02	0,22
SDM_mean					3,27	-0,70	0,98

Table S2: Standardized experimental data from DoE study of chromatography column 1 (CC1), as well as upper and lower normal operating ranges (NOR_U, NOR_L, respectively) and scale down model (SDM) variance and mean. Normalization was performed by subtracting all values by the mean and diving by the standard deviation of DoE runs.

Batches	Parameter End pooling	Parameter Elution strength	Paramet er wash strength	Parameter colum loading density	Parameter pH	Process impurity 2 clearance	Product impurity 2 clearance	Product impurity 1 clearance
DoE1	1.10	-1.10	-1.10	1.10	-1.10	-0.25	-0.55	-0.25
DoE2	0.00	1.10	-1.10	-1.10	-1.10	-1.07	-0.53	-0.75
DoE3	-1.10	0.00	-1.10	1.10	1.10	-0.34	-0.06	-0.36
DoE4	-1.10	-1.10	-1.10	-1.10	0.00	-0.51	2.72	-0.29
DoE5	1.10	-1.10	0.00	-1.10	1.10	0.33	NaN	NaN
DoE6	1.10	1.10	-1.10	0.00	1.10	-0.86	-0.28	-0.81

DoE7	-1.10	1.10	1.10	-1.10	1.10	-0.68	0.11	-0.62
DoE8	-1.10	1.10	0.00	1.10	-1.10	-0.65	-0.62	1.77
DoE9	0.00	0.00	0.00	0.00	0.00	0.86	-0.38	-0.78
DoE10	0.00	-1.10	1.10	1.10	1.10	0.75	1.21	0.44
DoE11	-1.10	-1.10	1.10	0.00	-1.10	0.79	-0.71	2.20
DoE12	1.10	0.00	1.10	-1.10	-1.10	-0.83	-0.48	0.06
DoE13	1.10	1.10	1.10	1.10	0.00	2.46	-0.42	-0.61
Threshold						-1.67	-1.04	-1.49
NOR_U	0.00	0.65	1.10	1.10	0.55			
NOR_L	-1.10	-1.10	-1.10	-0.51	-0.55			
Significant Parameters						Parameter wash strength, Parameter column loading density		Parameter end pooling, Parameter wash strength, Parameter column loading density, Parameter pH
SDM variance						0.03	0.00	0.02
SDM mean						-0.45	-0.58	-0.80
